# Antibody-Mediated Targeting of the Orai1 Calcium Channel Inhibits T Cell Function

**DOI:** 10.1371/journal.pone.0082944

**Published:** 2013-12-23

**Authors:** Jennifer H. Cox, Scott Hussell, Henrik Søndergaard, Kirstine Roepstorff, John-Vu Bui, Jen Running Deer, Jun Zhang, Zhan-Guo Li, Kasper Lamberth, Peter Helding Kvist, Søren Padkjær, Claus Haase, Stefan Zahn, Valerie H. Odegard

**Affiliations:** 1 Department of Cellular Immunology, Novo Nordisk Research Center, Seattle, Washington, United States of America; 2 Department of Immunopharmacology, Novo Nordisk A/S, Maløv, Denmark; 3 Department of Histology, Novo Nordisk A/S, Maløv, Denmark; 4 Department of Molecular Immunology, Novo Nordisk Research Center, Seattle, Washington, United States of America; 5 Department of Cell Biology, Beijing Novo Nordisk Pharmaceuticals Science & Technology Co., Beijing, China; 6 Department of Rheumatology & Immunology, Beijing University People’s Hospital, Beijing, China; 7 Department of Screening and Cell Technology, Novo Nordisk A/S, Maløv, Denmark; 8 Department of Protein Structure and Biophysics, Novo Nordisk A/S, Maløv, Denmark; 9 Department of Antibody Technology, Novo Nordisk A/S, Maløv, Denmark; University of Michigan Medical School, United States of America

## Abstract

Despite the attractiveness of ion channels as therapeutic targets, there are no examples of monoclonal antibodies directed against ion channels in clinical development. Antibody-mediated inhibition of ion channels could offer a directed, specific therapeutic approach. To investigate the potential of inhibiting ion channel function with an antibody, we focused on Orai1, the pore subunit of the calcium channel responsible for store-operated calcium entry (SOCE) in T cells. Effector T cells are key drivers of autoimmune disease pathogenesis and calcium signaling is essential for T cell activation, proliferation, and cytokine production. We show here the generation of a specific anti-human Orai1 monoclonal antibody (mAb) against an extracellular loop of the plasma membrane-spanning protein. The anti-Orai1 mAb binds native Orai1 on lymphocytes and leads to cellular internalization of the channel. As a result, T cell proliferation, and cytokine production is inhibited *in vitro*. *In vivo*, anti-Orai1 mAb is efficacious in a human T cell-mediated graft-versus host disease (GvHD) mouse model. This study demonstrates the feasibility of antibody-mediated inhibition of Orai1 function and, more broadly, reveals the possibility of targeting ion channels with biologics for the treatment of autoimmunity and other diseases.

## Introduction

Dysregulated T cell responses are a key driver of autoimmunity. Uncontrolled activation of self-reactive or microbial antigen–specific effector T cells, coupled with defects in the regulatory arm of the adaptive immune system, result in the breakdown of immune homeostasis and the development of immune-mediated diseases [1]. Calcium signaling is a requirement for T cell effector function and broad-spectrum calcium signaling inhibitors, such as cyclosporine A, inhibit inflammation in the clinic, but serious side effects limit their use [2]–[4]. The elucidation of the molecular composition of the channel required for calcium signaling in T cells presents an opportunity to develop a specific inhibitor of this calcium channel. Small molecule inhibitors and natural blocking agents, such as toxins, are commonly evaluated as ion channel blockers. Small molecule inhibitors, however, can lack target specificity given the highly homologous nature of ion channel families and toxins have a perceived risk of being immunogenic. A blocking monoclonal antibody, however, offers a more directed, virtually unexplored, therapeutic approach to inhibit ion channel function.

In T cells, store-operated calcium entry (SOCE) across the plasma membrane activates a signaling cascade that induces T cell effector functions such as proliferation and pro-inflammatory cytokine production. The calcium release activated channel (CRAC) formed by the protein Orai1 is responsible for SOCE in T cells [5]–. Function-abrogating genetic mutations in Orai1 have been defined in six patients [9], [10] all suffering from severe combined immunodeficiency syndrome (SCID) [11]–[13]. T cells isolated from these patients are defective in SOCE upon stimulation and are subsequently unable to produce inflammatory cytokines or proliferate. Likewise, T cells from mice lacking functional Orai1 are refractory to stimulation [14] and fail to induce colitis in an adoptive transfer model of inflammatory bowel disease [15].

Orai1 is a plasma membrane protein predicted to have four transmembrane segments and two extracellular loops of 20–40 amino acids in length. Upon engagement of the T cell receptor, release of intracellular calcium stored in the endoplasmic reticulum (ER) leads to the relocalization of Stromal Interaction Molecule 1 (STIM1) to the plasma membrane [16], [17] where it organizes Orai1 into a hexamer, forming an active calcium-selective channel [18], [19]. The resulting increase in intracellular calcium in turn activates transcription factors such as NFAT [20], [21]. Interestingly, patients with defects in STIM1 present with a similar clinical phenotype as those with defects in Orai1 [22].

Small molecule and natural toxin inhibitors directed against ion channels typically perturb function by binding or inserting into the channel pore. As is common within ion channel families, Orai1 exhibits a high degree of homology, over 90% identity, with its family members Orai2 and Orai3 in its transmembrane-spanning, or pore-forming, segments [23]. This high degree of homology presents a challenge in identifying a specific toxin or small molecule blocker. In contrast, the predicted extracellular region of Orai1, the area accessible to large molecules, is distinct in sequence from Orai2 and Orai3, providing an opportunity for identifying a molecule with a high degree of specificity. Despite ion channels such as Orai1 being key regulators of biological systems, there are currently very few examples of antibodies with specificity for this class of proteins in their native conformation and fewer examples still of antibodies capable of blocking channel function [24]–[27]. The very small extracellular regions of ion channels and the limited direct role of the extracellular region in channel function may render these challenging targets for a large molecule approach.

We report here the successful generation of a specific anti-Orai1 monoclonal antibody that inhibits T cell effector function *in vitro* and *in vivo* by reducing proliferation and pro-inflammatory cytokine production. We further utilized this antibody to characterize Orai1 expression on immune cell subsets from blood and rheumatoid arthritis synovial fluid. Our data demonstrate not only the therapeutic potential of antibodies targeting Orai1, but also highlight the underexplored opportunity of antibody-mediated blockade of ion channels for the treatment of disease.

## Materials and Methods

### Anti-Orai1 Antibody Generation and Purification

The peptide corresponding to the second extracellular loop (ECL2) of ORAI-1 (WVKFLPLKKQPGQPRPTSKPPASGAAANVSTSGITPGQA) was synthesized with an additional C-terminal cysteine and coupled to bovine serum albumin (BSA). Female eight week old RBF mice were immunized with ECL2-cBSA in complete Freund’s adjuvant. Splenocytes from mice with positive titers were fused by elecrofusion with the FOX-Ny myeloma cell line.

### ELISA Detection of Orai1-binding Antibodies

Culture supernatants from hybridomas were screened on Nunc immunoplates coated with 1 µg/mL of ECL2 peptide and blocked with PBS with 0.05% Tween20. Antibodies were detected with an HRP-labelled goat anti-mouse Fcγ secondary antibody (1 µg/ml), followed by development with TMB substrate (Kem-EN-Tec) as described by the manufacturer. Absorbance at 450 nm was measured.

### Binding of Anti-Orai1 to Transfectants and Primary Human Cells

Ba/F3 cells (DSMZ/RIKEN) were stably transfected with human Orai1 (Open Biosystems), Orai2 (Origene), or Orai3 (Origene) by electroporation. The Jurkat E6.1 cell line was transduced with (H)shRNA ORAI1 lentivirus particles (Santa Cruz Biotechnology) following manufacturer’s procedures. Stable clones were assayed for Orai1 expression by qPCR. Anti-Orai1 or mIgG1 control were incubated with cells, and then detected with a fluorophore-conjugated goat anti-mouse IgG. Cells were analyzed on the LSRII flow cytometer (Becton Dickinson) and analysis was completed using Tree Star’s FlowJo analysis software. PBMCs were isolated from apheresis units from healthy donors with written informed consent and study approval by the New England Institutional Review Board (Research Blood Components; Boston, MA). Binding was analyzed as above, including cell surface antibodies to: CD3, CD4, CD8, CD45RA, CD45RO, CD19, CD20, IgD, CD27, CD14, CD56, CD86, CD11c, and HLA-DR.

### In vitro Functional Assays

#### Calcium flux

Jurkat cells, calcium starved in HBSS lacking Ca^2+^ and Mg^2+^ (Gibco), were plated at 300,000 cells per well in 96-well Optilux plates (BD Pharmingen). Anti-Orai1 or mIgG1 control antibodies and FLIPR Calcium 4 no-wash reagent (Molecular Devices) were added for 1 hour at 37°C. Final concentrations of 1 µM thapsigargin (Sigma) and 2 mM Ca^2+^ were added by the Flexstation 3 (Molecular Devices) and fluorescence was read at 485/530 nm.

#### Internalization assay

Prior to experiment, anti-Orai1 mAb was conjugated to Alexa Fluor 647 dye (Molecular Probes/Life Technologies) and anti-Cy5 mAb (clone CY5-15; AbCam) was biotinylated using EZ-Link NHS-PEG4-Biotin (Thermo Scientific). CD4^+^ T cells were isolated from apheresis units (StemCell Technologies). Cells were diluted in RPMI 1640 containing Glutamax, 25 mM Hepes, and 10% heat inactivated FBS. 1×10^5^ cells/well plated in 96 well U-bottom plates (BD FALCON) were allowed to equilibrate to either 4°C or 37°C. Anti-Orai1-AF647 (2 µg/mL) was incubated for 30 and 60 minutes at the appropriate temperature. Cells were washed with ice cold PBS/5% heat inactivated FBS then fixed for 10 minutes with 4% PFA. Biotinylated anti-Cy5 (10 µg/mL) & anti-CD4-PE (1∶200, eBioscience) were added for 1 hour at room temperature, followed by SA-BV421 (1∶1000, Biolegend) for 30 minutes at room temperature. Cells were analyzed by flow cytometry as previously mentioned.

#### Anti-CD3/Anti-CD28 Stimulated PBMC Proliferation

PMBCs were CFSE-labeled (CellTrace; Invitrogen) following manufacturer’s instructions. Antibodies and cyclosporine A (Sigma) were added to 200,000 cells per well in 96-well U-bottom plates and incubated 1 hr at 37°C in 5% CO_2_. Anti-CD3, UCHT1 (1 ng/mL) and anti-CD28, CD28.2 (1 µg/mL) (eBioscience) antibodies were added and incubated for 3 days. Cells were labeled with Live/Dead® Fixable Aqua Dead Cell Stain (Invitrogen) and CFSE dilution was measured on a LSRII. Supernatants were removed at 16 and 72 hours for IL-2 and IFN-γ measurements by Millipore Immunoassay.

#### Staphylococcal Enterotoxin B (SEB) assay

Frozen human RA patient PBMCs (Astarte Biologics) were CFSE-labeled as above. Antibodies were incubated with 100,000 cells per well for 1 hour at 37°C then 1.25 ng/mL SEB (Sigma) was added. Cells were incubated 6 days and stained with Live/Dead® Fixable Far Red Dead Cell Stain and CFSE dilution was analyzed.

#### Tetanus toxoid assay

PMBCs were CFSE-labeled as above. Antibodies and cyclosporine A (Sigma) were added to 100,000 cells per well of 96-well U-bottom plate and incubated for 1 hour as above. Tetanus toxoid (Calbiochem) was added at 0.125 µg/mL and incubated for 5 days. Cells were labeled with Live/Dead® stain and CFSE dilution was measured.

### Immunohistochemical Detection of Orai1 in Synovial Tissue

Formalin-fixed, paraffin-embedded synovial tissue samples from RA (n = 24) or HC (n = 11) were obtained from Cambridge Biosciences (Cambridge, UK). Sections were blocked in 3% skim milk, 7% donkey serum, and 3% human serum in TBS followed by incubation with 0.05 µg/ml of rabbit anti-Orai1 O8264 (Sigma Aldrich, St. Louis, MO) at 4°C overnight. Slides were incubated with biotin-conjugated donkey anti-rabbit secondary antibodies, then by peroxidase-conjugated avidin–biotin complex (VectorStain, Vector Laboratories) for and finally by indirect biotin-conjugated tyramide signal amplification systemand peroxidase-conjugated avidin–biotin complex (VectaStain) (Vector Laboratories) and developed with DAB chromogen before counterstaining with hematoxylin. All slides were scanned in a NanoZoomer 2.0 HT slide scanner (Hamamatsu Photonics) and a semi-quantitative scoring system ranging from 0–4 was used: 0 = no Orai1-positive cells, 1 = a few Orai1-positive cells, 2 = some Orai1-positive cells, 3 = several Orai1-positive cells, and 4 = many Orai1-positive cells.

### Rheumatoid Arthritis Synovial Fluid Experiments

Fresh synovial fluid samples were obtained from two symptomatic female RA patients. The study was approved by the medical ethics committee of Peking University People’s hospital and written informed consent was obtained from all participants. Synovial cells were incubated with 10 µg/mL anti-Orai1 or isotype control in FACS buffer (DPBS with 0.1% NaN_3_ and 0.1% BSA) for 20 minutes at room temperature, followed by a PE-conjugated rat anti-mouse IgG1 (eBioscience) for 15 minutes at room temperature. Cells were stained with antibodies to: CD4, CD8, CD14, CD19, CD66b (BD Biosciences/eBiosciences). For cytokine production assays, 96-well U-bottom plates were pre-coated with 0.3 µg/mL anti-CD3 HIT3a (BD Pharmingen) and 3 µg/mL anti-CD28.2 (BD Pharmingen). Indicated treatments were incubated with 1×10^5^ cells per well for 40 hours in RPMI 1640, 10% heat inactivated FBS, 1% penicillin/streptomycin. Supernatants were analyzed for IL-2 and IFN-γ by ELISA (eBioscience).

### Humanized GvHD Model

#### Humanized mice

All animal experiments were approved by Novo Nordisk’s internal Ethical Review Council as well as the Danish Animal Inspectorate (license number 2009/561–1673). Female NOD.scid (IL-2Rγc)^−/−^ (NOG) mice (Taconic) were injected i.v. with 20×10^6^ human PBMCs from healthy donors. Antibodies were administered on day 0 by i.p. (10 mg/kg) and 3 times per week thereafter. Mice experiencing more than 20% weight loss or impaired general health were euthanized by cervical dislocation or CO2.

#### FACS analysis of splenocytes

Following onset of GvHD, splenocytes were stained with 10 µg/mL anti-Orai1 or mIgG1 isotype control (R&D Systems) followed by 1∶100 dilution of F(ab’)2 Fragment Goat Anti-Mouse IgG-APC (Jackson ImmunoResearch) Antibodies to cell surface markers were added, mCD45 (Caltag), hCD3 (Invitrogen), hCD45, hCD4, hCD8 (all BD Pharmingen), and LIVE/DEAD® Fixable Near-IR Dead Cell Stain.

#### FACS analysis of blood samples

Blood samples were taken under isoflurane anaesthesia weekly throughout the study and at time of GvHD onset. RBCs were lysed and FcR were blocked (Fc Block, BD Biosciences). Surface staining was done using anti-human CD45, CD4, CD8, CD19 (all BD Biosciences), CD3 (Invitrogen), and anti-mCD45 (Caltag), LIVE/DEAD® Fixable Near-IR Dead Cell Stain Kit. Cells were transferred to BD TruCount tubes (BD Biosciences) for absolute cell counting and analyzed by FACS.

#### Human IFN-γ detection in plasma by ELISA

Plasma samples were analyzed for human IFN-γ using a Human IFN-γ ELISA Ready-SET-Go reagent set (eBiosciences).

#### Immunohistochemical staining and quantification of human CD8^+^ cells in murine liver and lungs

Liver and lung tissue from euthanized mice were formalin-fixed and paraffin-embedded. Tissue sections were incubated overnight at 4°C with either 0.5 µg/mL polyclonal rabbit anti-human CD8 antibody (M3169) or polyclonal control in a TBS buffer (7% goat and 3% mouse serum, 3% BSA and 0.5% skim milk). Secondary antibody-polymer complex (Envision, K4003) was applied for 30 min at room temperature. Slides were developed with di-amino benzidine (DAB) and counterstained in Meyer’s haematoxylin. All sections were scanned using the Nanozoomer 2.0 HT system (Hamamatsu, Glostrup, Denmark). Automated image analysis was performed on scanned immunostained images with the Visiopharm Integrator System (version 4.2.2.0, Visiopharm, Hørsholm, Denmark). Tissue detection was performed using the Visiomorph DP module allowing the generation of a region of interest (ROI) around the tissue. All data were generated by doing batch analysis in the VIS software.

### Statistics

Data were analysed using Prism software (Graphpad Software, Inc) by Student’s *t* test. In addition, Kaplan-Meier survival analysis and Mantel-Cox Log-Rank test was used in analysis of GvHD and Mann-Whitney’s U-test was used in analysis of human T cells in blood. Bar plots show mean ± SEM and a p-value <0.05 was considered statistically significant, *p<0.05, **p<0.01, ***p<0.001.

## Results

### Generation of Monoclonal Antibody with Specificity for Native hOrai1

**Figure 1:**
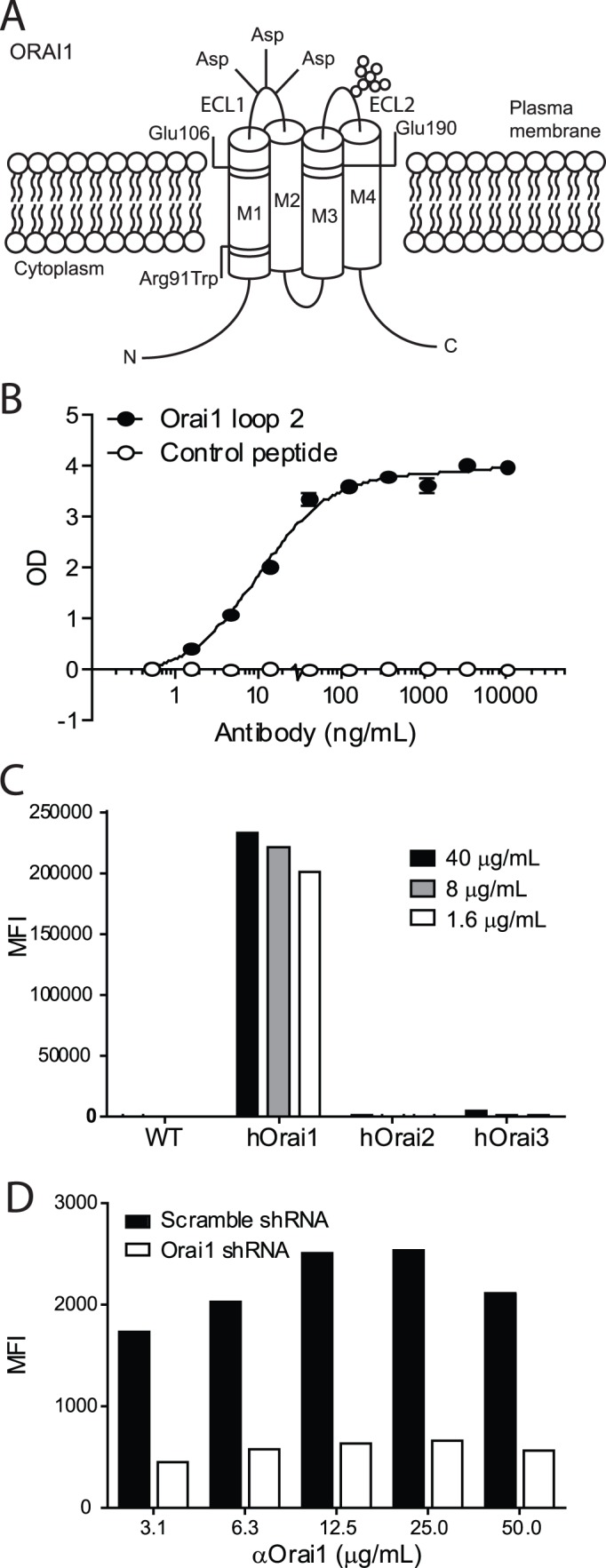
Generation of specific anti-human Orai1 monoclonal antibody. **A**) Representation of human Orai1 tetra-membrane spanning protein with two extracellular loops of approximately 20 and 40 amino acids, respectively. **B**) Titration of purified monoclonal anti-Orai1 antibody binding to loop 2 peptide in ELISA assay. **C**) Ba/F3 cells overexpressing human (h)Orai1, Orai2, or Orai3 or **D**) Jurkat cells transduced with Orai1-targeting shRNAi or scramble control were incubated with purified anti-Orai1 at the indicated concentrations and detected with fluorophore-conjugated goat anti-mouse IgG. MFI indicates median fluorescence intensity. The experiment in panel B was performed in duplicate and is representative of three individual experiments. Panel C is from single wells from one experiment and panel D is from single wells and is representative of two individual experiments.

There are two predicted extracellular loops, ECL1 and ECL2, in human Orai1 as depicted in Fig. 1A. BALB/c mice were immunized with peptides spanning either the first or second extracellular loops of human Orai1. A protein BLAST search confirmed that hOrai1 is the only protein with identity to the peptides. While both peptides gave rise to polyclonal titers reactive to the respective immunizing peptide, only the peptide spanning ECL2 gave rise to titers that recognize native Orai1 protein on the surface of cells (data not shown) and efforts to generate antibodies recognizing the ECL1 of native Orai1 (including whole cell immunizations, varying mouse strains and adjuvant usage) were not successful.

As shown in Fig. 1B, we identified an Orai1 reactive clone, 10F8, that binds to the immunizing peptide by ELISA and to a Ba/F3 cell line engineered to over-express Orai1, demonstrating that 10F8 can recognize hOrai1 expressed on the cell surface. 10F8 does not bind to Ba/F3 parental cells or Ba/F3 cells transfected with closely-related family members hOrai2 or hOrai3 (Fig. 1C). Antibodies specific for Orai2 and Orai3 do not exist, so expression was confirmed by RT-PCR and detection of a co-expressed FLAG tag in transfected Ba/F3 cells (data not shown). The Jurkat T cell line expresses endogenous Orai1 that can be detected by 10F8. Orai1-targeted shRNAi was used to generate Jurkat clones with negligible Orai1 expression as determined by RT-PCR (data not shown). The specificity of 10F8 for Orai1 was confirmed by its reduced binding to the Orai1 knockdown lines as compared to the scramble shRNAi control Jurkat lines (Fig. 1D).

### Cell Surface Expression of Orai1 on Primary Human Immune Cells

**Figure 2:**
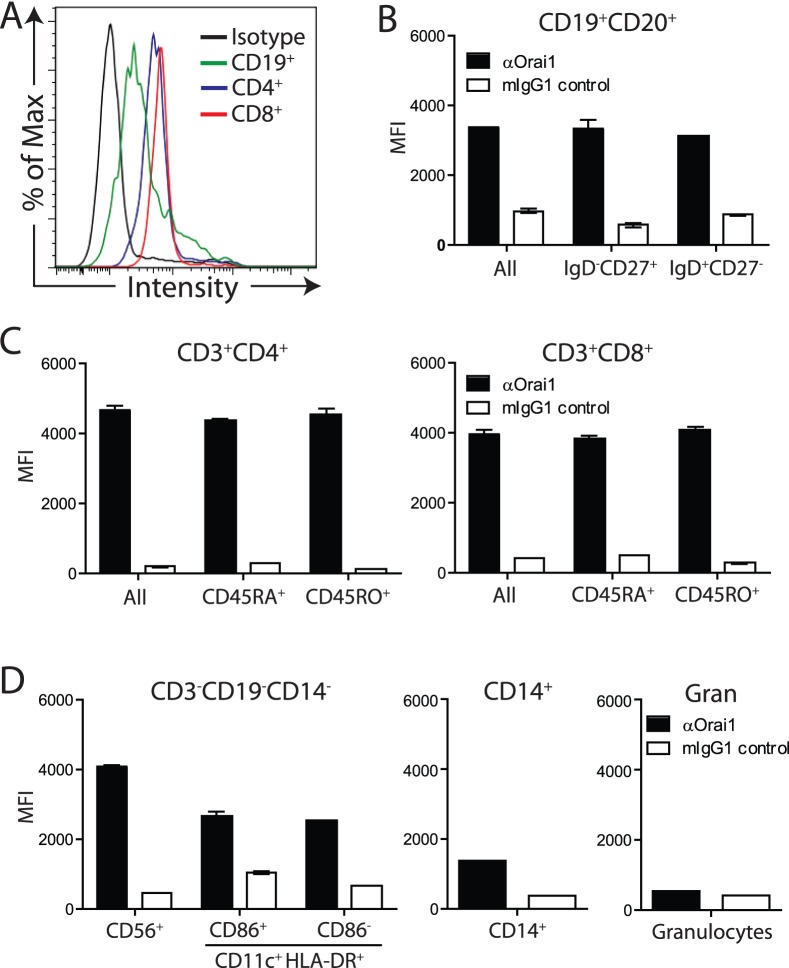
Anti-Orai1 antibody binds to native Orai1 on primary immune cells. **A**) Representative histogram demonstrating αOrai1 or isotype control binding to primary immune cells (CD3^+^CD4^+^, CD3^+^CD8^+^, and CD19^+^) from the peripheral blood of a healthy individual using 12.5 µg/mL antibody. **B**) Median Fluorescence Intensity (MFI) of αOrai1 and mIgG1 control binding to memory (IgD^−^CD27^+^) and naïve (IgD^+^CD27^−^) CD19^+^ B cells; antibody used at 12.5 µg/mL. **C**) Binding of αOrai1 and mIgG1 control to naïve (CD45RA^+^) and memory (CD45RO^+^) CD3^+^CD4^+^ and CD3^+^CD8^+^ T cells. **D**) Binding of aOrai1 to non-lymphocyte NK cells (CD3^−^CD19^−^CD14^−^CD56^+^), CD86^+^ and CD86^−^ dendritic cells (CD3^−^CD19^−^CD14^−^CD11c^+^HLA-DR^+^), CD14^+^ monocytes, and granulocytes. All data is representative of at least three independent donors.

The generation of an Orai1-specific antibody provides the first opportunity to characterize cell surface expression of Orai1 on peripheral immune cells. PBMC isolated from healthy donors were stained with 10F8 and, consistent with the T, B, and NK cell defects described in humans carrying loss-of-function mutations in Orai1, Orai1 is detectable on both CD4^+^ and CD8^+^ T cells, CD56^+^ NK cells, and, to a lesser extent, CD19^+^ B cells (Fig. 2A). Amongst B and T cells, there is no significant difference in Orai1 cell surface expression between memory and naïve subsets (Fig. 2B, 2C). CD86^+^ or CD86^−^ dendritic cells have a moderate level of expression and expression is low on CD14^+^ monocytes (Fig. 2D). In contrast, we are unable to detect Orai1 on granulocytes (Fig. 2D). These data suggest that Orai1 may play a role in regulating dendritic cell and monocyte function, in addition to its reported role in lymphocytes.

### Anti-Orai1 Monoclonal Antibody Inhibits T cell Responses in vitro

**Figure 3:**
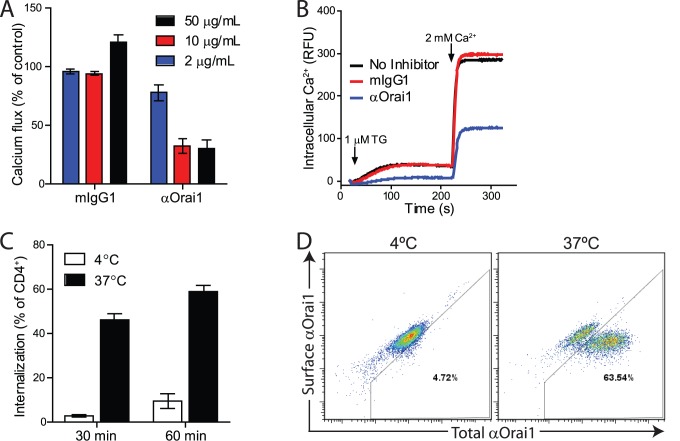
Anti-Orai1 antibody inhibits calcium flux and induces internalization in T cells. **A**) Inhibition of thapsigargin-induced calcium flux in Jurkat cells with αOrai1. Data is calculated as percentage of ΔRFU following calcium influx in the absence of antibody addition. **B**) Representative trace of thaspigargin (TG)-induced calcium influx in Jurkat cells treated with 50 µg/mL αOrai1 or mIgG1 control. **C**) αOrai1 internalization in purified human CD4^+^ cells was measured following incubation at 37°C by flow cytometry using 2 µg/mL αOrai1-AF647 and cell surface detection with biotinylated anti-Cy5 followed by streptavidin-BV421. Samples at 4°C were analyzed in parallel as negative controls for internalization. Data is calculated as percentage of total CD4^+^ cells and is the average of three donors. **D**) Representative flow cytometry plots of surface bound αOrai1 compared to total internalized and surface αOrai1. Panels A and B are representative of three independent experiments and panels C and D are representative of two experiments.

Since previous studies show that Orai1 is critical for calcium entry into Jurkat T cells, we used Jurkat cells to confirm that 10F8 is a functionally blocking antibody. Indeed, thapsigargin-induced calcium flux is specifically inhibited by the anti-Orai1 antibody (Fig. 3A, 3B). Our data suggest that antibody-mediated internalization of Orai1 contributes to the observed functional inhibition as 10F8 can induce Orai1 internalization in primary T cells (Fig. 3C, 3D). Together, these data support the use of 10F8 for investigating the role of Orai1 in effector function of differentiated immune cells.

**Figure 4:**
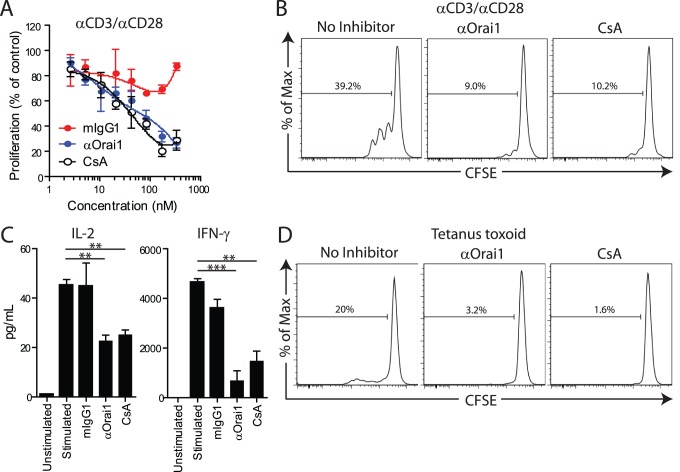
Anti-Orai1 antibody inhibits T cell response and proliferation. **A**) Proliferation of PBMCs treated with αCD3/αCD28 in the presence of control mIgG1, αOrai1, or cyclosporine A (CsA). Data is calculated as percentage of CFSE-diluted cells in the absence of inhibitor. **B**) Representative CFSE dilution traces of αCD3/αCD28-treated PBMCs without inhibitor, or with 333 nM CsA or αOrai1 (equivalent to 50 µg/mL). FACS plots are gated on viable CD5^+^ T cells. **C**) Effect of treatment with 20.8 nM CsA or αOrai1 (equivalent to 3.1 µg/mL) on IL-2 and IFN-γ production at 16 and 72 h, respectively, following stimulation with αCD3/αCD28. **D**) Representative CFSE dilution traces of tetanus toxoid-induced proliferation of PBMCs. FACS plots are gated on viable CD3^+^CD4^+^ T cells. **P<0.01, ***P<0.001. Panels A–C are representative of three independent experiments (each with at least two donors) and panel D is from a single experiment.

We analyzed the effects of anti-Orai1 mAb treatment on T cells from healthy donors. Total PBMCs were stimulated with αCD3/αCD28 in the presence of anti-Orai1 mAb, isotype control, or the broad-spectrum calcium signaling inhibitor, cyclosporine A. The anti-Orai1 mAb significantly attenuates T cell proliferation, as measured by CFSE dilution, comparable to or better than molar equivalent concentrations of cyclosporine A (Fig. 4A, 4B). IL-2 and IFN-γ production are also reduced in the presence of anti-Orai1 mAb at the 16 and 72 hour timepoints, respectively (Fig. 4C). We also tested the requirement for Orai1-mediated calcium flux in antigen-specific memory T cell responses. PBMCs isolated from donors previously immunized with tetanus antigen were incubated with tetanus toxoid in the presence or absence of anti-Orai1 mAb. T cell proliferation is reduced upon treatment with anti-Orai1 mAb, comparable to effects observed with cyclosporine A treatment (Fig. 4D). These data show that an Orai1-blocking mAb is effective at preventing polyclonal and antigen-specific T cell responses *in vitro.*

### Anti-Orai1 mAb Inhibits Cytokine Production from Immune Cells Isolated from Rheumatoid Arthritis Patients

**Figure 5:**
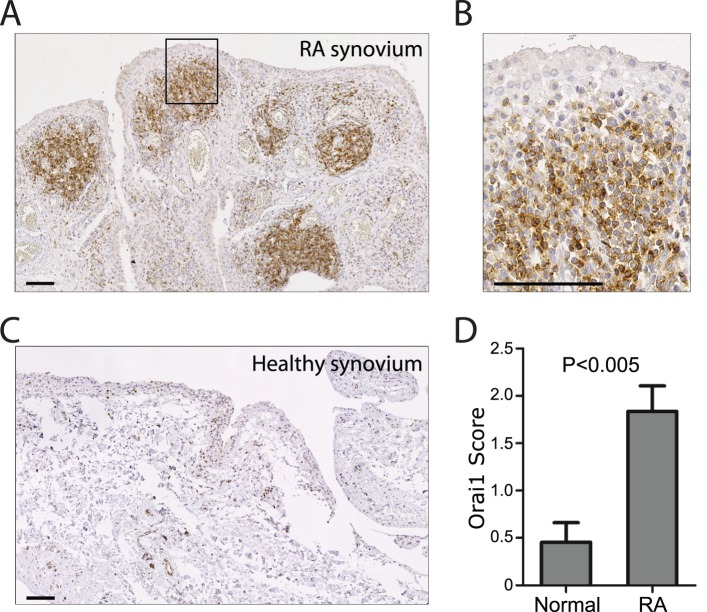
Orai1 expressing cells are abundant in synovial tissue from patients with rheumatoid arthritis. Sections of synovial tissue from patients with rheumatoid arthritis or healthy controls were stained for Orai1 by immunohistochemistry with rabbit anti-Orai1 (Sigma), and the number of Orai1-positive immune cells was evaluated by assigning each stained tissue section a semi-quantitative score ranging from 0–4. **A**) Orai1 immunohistochemistry of rheumatoid arthritis synovial tissue. **B**) Magnification of A. **C**) Orai1 immunohistochemistry of normal synovial tissue. **D**) Histogram showing mean semi-quantitative scores of the number of Orai1-positive cells in synovial tissue from rheumatoid arthritis patients (RA) (n = 24) or normal synovial tissue (n = 11). Error bars, +/− SEM. Scale bars, 100 µm.

**Figure 6:**
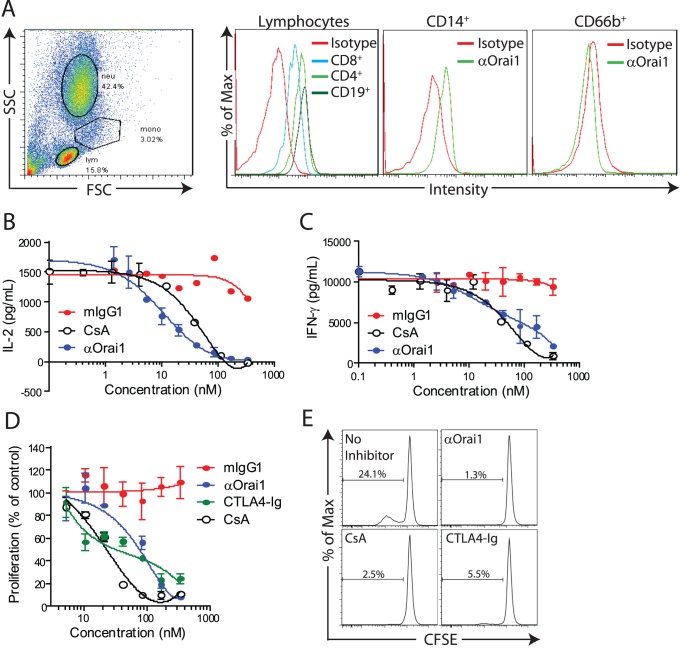
Anti-Orai1 reduces cytokine production by RA synovial fluid cells. **A**) Representative forward and side scatter of synovial fluid cells (SFCs) with gating of lymphocytes (lym), monocytes (mono), and neutrophils (neut) and surface staining of Orai1 on CD4^+^, CD8^+^, and CD19^+^ lymphocytes, CD14^+^ monocytes, and CD66b^+^ neutrophils from RA synovial fluid. **B**) IL-2 and **C**) IFN-γ secretion from αCD3/αCD28 costimulated SFCs following 40 hour-culture in the presence of mIgG1 isotype control, αOrai1, or cyclosporine A (CsA). **D**) Proliferation of RA patient PBMCs treated with SEB in the presence of control mIgG1, anti-Orai1, CTLA4-Ig, or cyclosporine A (CsA). Data is calculated as percentage of CFSE-diluted cells in the absence of inhibitor. **E**) Representative CFSE dilution traces of SEB-induced proliferation of RA PBMCs in the presence of 333 nM inhibitor. All panels are representative of two independent experiments.

The synovial membrane in patients with rheumatoid arthritis (RA) is characterized by an infiltrate of inflammatory cells, primarily CD4^+^ T cells [28]. To validate the relevance of Orai1 in RA pathology, synovial tissue from rheumatoid arthritis patients was assessed for Orai1 expression by immunohistochemistry using a commercially available polyclonal antibody. While abundant staining is observed in RA tissue (Fig. 5A, 5D), healthy controls show minimal levels of Orai1 expression (Fig. 5C, 5D). More evident at higher magnification, the focal staining and cellular morphology of Orai1 positive cells is consistent with infiltrating immune cells (Fig. 5B). Similarly, the anti-Orai1 antibody binds to CD4^+^ and CD8^+^ T cells as well as CD19^+^ B cells in RA synovial fluid (Fig. 6A). In contrast, no binding is detected on CD66b^+^ granulocytes and modest expression is found on CD14^+^ monocytes (Fig. 6A). When synovial fluid mononuclear cells are stimulated with αCD3/αCD28, anti-Orai1 mAb effectively inhibits both IL-2 and IFN-γ secretion to a level comparable to or better than that achieved with cyclosporine A (Fig. 6B, 6C). These data show that Orai1 controls the effector function of T cells at the site of inflammation in RA.

To confirm that Orai1 is also critical for controlling the function of peripheral T cells in patients with ongoing autoimmune disease, we stimulated PBMCs isolated from RA patients with the superantigen SEB (Staphylococcus aureus enterotoxin B). The induction of T cell proliferation in this system requires endogenous costimulation through CD28 and can be blocked by the addition of CTLA4-Ig, a therapeutically effective molecule in RA. In this system, T cell proliferation is similarly inhibited by the addition of either anti-Orai1 mAb or CTLA4-Ig (Fig. 6D, 6E), albeit through targeting distinct mechanisms. These data further support the targeting of Orai1 as a therapeutic strategy to inhibit overactive T cell responses in autoimmune disease.

### In vivo Efficacy of Anti-Orai1 in Humanized GvHD Model

**Figure 7:**
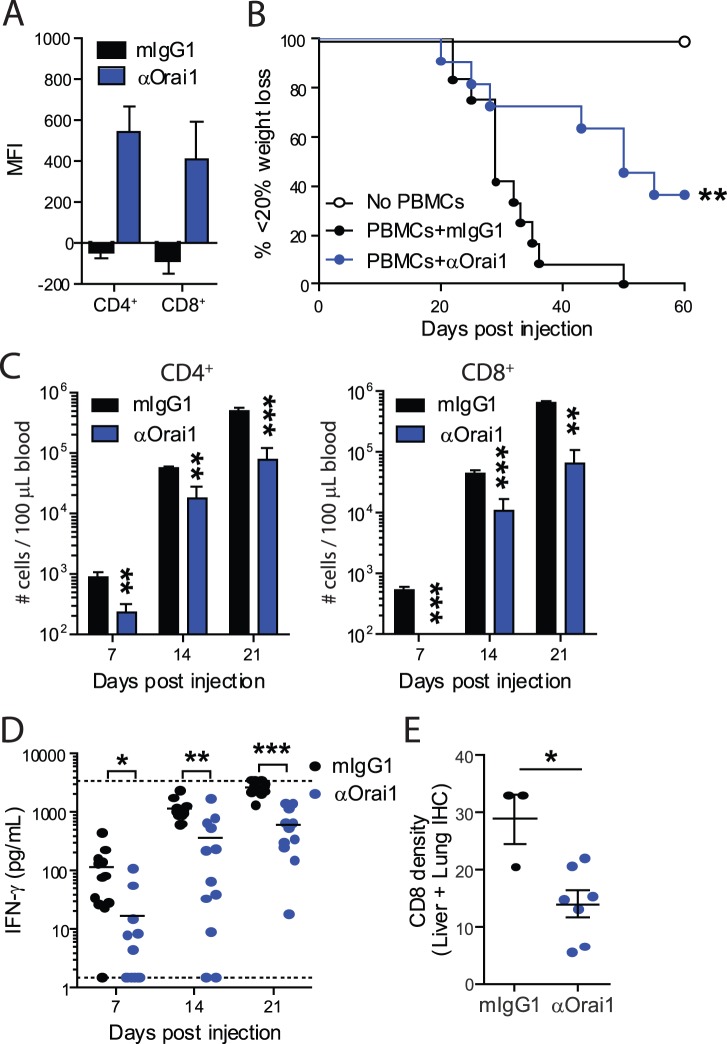
Anti-human Orai1 antibody attenuates xenogeneic GvHD in humanized mice. Groups of humanized NOG mice were treated with 10/kg αOrai1 (N = 11) or mIgG1 isotype control (N = 12) 3 times per week throughout the study. Mice without PBMC transfer were included as controls (N = 3). **A**) Staining of Orai1 on human CD4^+^ and CD8^+^ T cells from spleens of humanized mice following the onset of GvHD. **B**) Kaplan-Meier curves depict percentage of mice without GvHD defined as <20% weight loss. **C**) Absolute numbers of human CD4^+^ and CD8^+^ T cells in blood were quantified by flow cytometry on indicated days post PBMC injection. **D**) Human IFN-γ in plasma was measured by ELISA where horizontal dotted lines indicate assay range. **E**) Paraffin-embedded sections of livers and lungs were immunohistochemically stained with anti-human CD8 and analyzed using automated image analysis software (VIS) to show the CD8 density (% tissue area stained with CD8). Data are individual or mean +/− SEM and representative of two separate experiments. **P<0.01 by Mantel-Cox Log-Rank test compared to PBMCs+mIgG1 (B). *p<0.05, **P<0.01, ***P<0.001 by Mann-Whitney U-test (B-D). In (D), samples above range were given the maximum assay value and included in statistics whereas values below detection were plotted with the value 1, but not included in statistics.

In light of the significant T cell inhibition achieved *in vitro* with the anti-Orai1 mAb, we next wanted to evaluate the efficacy of this antibody in a T cell-mediated mouse disease model. Since the anti-Orai1 mAb does not cross-react with rodent Orai1 (data not shown), we chose to use a mouse model of Graft-Versus-Host Disease (GvHD) in which human PBMCs are transferred into immunodeficient NOD.scid.IL-2Rγc^−/−^ mice, hereafter referred to as the humanized GvHD model [29], [30]. In this model, human T cells engraft, expand and cause multi-organ inflammation (GvHD) within 30–45 days post-transfer. Disease progression can be followed by physical signs of GvHD (weight loss), human T cell expansion in the blood, and by histological analysis of organ inflammation post-mortem. The 10F8 antibody was used to confirm Orai1 expression on human CD4^+^ and CD8^+^ T cells isolated from the spleens of mice at the time of disease onset (Fig. 7A). Thus, this humanized GvHD model offers the opportunity to study the effects of an anti-human Orai1 antibody *in vivo* on human T cell expansion and human T cell-mediated GvHD. As shown in Fig. 7B, anti-Orai1 mAb significantly delays the time to and incidence of GvHD measured by weight loss, compared to the isotype control. Treatment with the anti-Orai1 mAb reduces both human CD4^+^ and CD8^+^ T cell numbers in blood compared to the isotype control at every time point analysed (Fig. 7C). Yet, substantial T cell expansion was observed following anti-Orai1 treatment, suggesting reduced T cell expansion by anti-Orai-1 mAb treatment rather than T cell depletion. Furthermore, anti-Orai1 mAb treatment reduces IFN-γ levels in plasma (Fig. 7D) and the density of infiltrating CD8^+^ cells in the lungs and liver (Fig. 7E), relative to mice treated with isotype control. These data clearly demonstrate the efficacy of antibody-mediated Orai1 targeting *in vivo*.

## Discussion

Despite remarkable growth in the development of therapeutic antibodies in recent years, there remain only limited examples of antibodies targeting ion channels [24]–[26]. In autoimmune disease, several ion channels have the potential to be high value therapeutic targets. For example, the Orai1 calcium channel and Kv1.3 potassium channel both have substantial genetic and biological data supporting therapeutic blockade [31]. Small molecule inhibitors have been generated for multiple ion channels, including Orai1, but challenges due to poor specificity and the associated risk of off-target effects can slow progress towards the clinic. Lin et al. recently reported anti-Orai1 antibodies that inhibit *I*_CRAC_ currents in overexpressing cell lines and cytokine production in whole blood [27]. Here we describe an independently generated anti-Orai1 antibody, with specificity for ECL2, which inhibits T cell activation *in vitro* and T cell mediated GvHD *in vivo*.

To generate anti-Orai1 antibodies, immunization strategies targeted both the first and second extracellular loops of human Orai1, approximately 20 and 40 amino acids in length, respectively. Antibodies specific for ECL2 were obtained by using either peptide-conjugates or overexpressing cell lines as immunogens. In contrast, despite employing a number of different immunization strategies, we could not raise antibodies to ECL1. The small size of the first loop and the relatively high homology between human and mouse amino acid sequence in this region could contribute to the difficulties in raising antibodies to ECL1. It is also conceivable that the native structure of Orai1 renders ECL1 inaccessible to antibody binding. ECL1 is involved in ion selectivity [32], [33] and in mediating the transition between open and closed channel states [34], but a functional role for the ECL2 has not been described. The data presented here suggest that the antibody against ECL2 inhibits Orai1 function by internalizing the Orai1 channel with an associated reduction in calcium flux, the first time to our knowledge that an anti-ion channel antibody has been shown to have such function. The Orai1 antibody described herein will aid in studying the expression and function of native Orai1-containing calcium channels. Studies with cells isolated from individuals carrying inactive genetic variants of Orai1, while informative, have not allowed for delineation of the function of Orai1 in immune cells during development as opposed to after differentiation. It is conceivable that lack of functional Orai1 during development triggers compensatory mechanisms, such as a shift towards using other calcium channels that could skew functional analysis. SOCE is induced upon receptor engagement in a number of immune cell types, including lymphocytes, mast cells, dendritic cells, neutrophils and macrophages [6]. While studies of Orai1 function in the human immune system have largely focused on lymphocytes, Orai1 deficient mice have been used to demonstrate a requirement for Orai1 in granule-release by mast cells [35]. Furthermore, RNAi studies in a neutrophil cell line have suggested a role for Orai1 in the regulation of neutrophil motility [36], although Orai1 was undetectable on the surface of neutrophils in our studies. The surface expression analysis presented here also highlights the importance of addressing the function of Orai1 in myeloid cell types in future studies.

Human genetic studies suggest that Orai1 is a promising therapeutic target for the treatment of autoimmune diseases mediated by dysregulated T cell responses and Orai-1 is expressed in synovial fluid cells and tissue from RA patients. Blockade of calcium signaling with broad spectrum inhibitors such as cyclosporine A and tacrolimus has been efficacious in severe autoimmune patients, but these treatments are often associated with use-limiting toxicities. The generation of a specific anti-Orai1 monoclonal antibody offers a more directed approach for inhibiting calcium signaling in T cells. Indeed, the anti-Orai1 mAb is effective in preventing T cell proliferation and cytokine release *in vitro* and is efficacious in T cell-mediated GvHD *in vivo*. However, treatment with any immunosuppressive therapy also faces the risk of increased susceptibility to opportunistic infections. Taken together, these data support further development of antibodies for the blockade of Orai1, as well as other ion channels implicated in immune cell function, as a novel treatment strategy for autoimmune disease.
